# From frozen to feeding: storage characteristics of banked donor human milk used in a single level IV academic neonatal intensive care unit

**DOI:** 10.21203/rs.3.rs-4486977/v1

**Published:** 2024-06-13

**Authors:** Katherine Chetta, Mary Galemmo, Terence Camilon, Wrenn Tiernan, Whitney Savino, Allison Rohrer, John Baatz, Carol Wagner

**Affiliations:** Medical University of South Carolina; Medical University of South Carolina; Medical Univ South Carolina; Medical University of South Carolina

**Keywords:** donor human milk, preterm infant, nutrition, breast milk storage, neonatal intensive care unit

## Abstract

**Background:**

The storage time of banked donor human milk (DHM) administered in an academic hospital to critically ill preterm neonates was previously unknown.

**Objective:**

This study was designed to determine the storage time of banked DHM by measurements obtained at the hospital level (by lot finish date) and individual patient level (by feeding date) over 2-year observation period.

**Results:**

Both methods of measuring storage time (hospital-level and patient-level) showed that DHM was stored on average 8 ±1 months before use. Variations in storage time fluctuated across months with a minimum and maximum storage duration of 119 to 317 days. Most infants received a median of 3 [2–5 IQR] unique lots of DHM.

**Conclusion:**

The storage time of DHM was successfully measured. Over 95% of DHM received was stored longer than 6 months. Storage times varied widely, uncovering a potential area of future research.

## Introduction

Human milk is the preferred enteral nutrition for the preterm infant by lowering the risk of necrotizing enterocolitis (NEC), improving growth, and protecting neurodevelopment. Banked DHM is recommended by the American Academy of Pediatrics when mother’s milk is not available, and DHM is most efficacious for the very low birth weight population (those born less than 1500 g, VLBW). Frozen human milk can be stored up to 12 months before use, according to the CDC (1). The Human Milk Banking Association of North America (HMBANA) follows this 12-month limit, yet the length of time that banked DHM is stored before hospital receipt is not known. No studies have reported the storage of banked DHM used in a U.S neonatal intensive care unit (NICU). When milk is stored, changes in the bioactivity of key proteins, lipids, and antioxidants can occur (2–5). Characterizing the magnitude and effect of variations in these bioactive compounds on outcomes could be a promising area of translational research. The aim of this study was to determine the estimated storage time of DHM at both the hospital-level and patient-level in a single center. We describe two methods to ascertain DHM storage time as a novel research variable.

## Methods

This was a prospective 2-year cohort study performed February 2022 to February 2024, designed to characterize donor human milk lots used in a large academic 82-bed level IV NICU. Two approaches were used to characterize banked DHM received by Shawn Jenkins Children’s Hospital, a high-volume, free-standing children’s hospital. Recorded variables were determined by the nutrition management center (“milk room”) technicians. VLBW infants were selected to prospectively follow for up to 21 days if they were born between 2022–2024 and received donor human milk. They were excluded if they never fed, died before 21 days, had major life-threatening anomalies, or were transferred in > 24 hours of life. This study was approved by the Medical University of South Carolina Institutional Review Board under study protocol#104755 (VLBW Nutrition and Growth Study) for which consent was waived.

### Data sources

All DHM lots were sourced from a local HMBANA-certified non-profit milk bank that adheres to standard guidelines including storing milk over 12 months in the freezer, pooling 3–5 donors, assessing caloric content, and performing Holder pasteurization with adjunct post-pasteurization microbial testing (6). Over the study period, the order date of DHM, lot expiration date, and the date that each lot was completed (finish date) were recorded. Unique DHM bottles and feeding events were recorded in the hospital-based Timeless^™^ Medical Women and Infants system for breast milk tracking, using a patient’s unique mom and baby ID number. Electronic medical record data by EPIC^®^ was used to verify DHM receipt. DHM packing slips contained the lot (or batch) expiration date.

#### Method 1: Calculating monthly storage times by hospital receipt of lots (hospital-level).

Lot numbers, number of bottles per lot, and lot expiration dates were recorded by hospital milk room technicians. The ‘order-date’ was recorded as the general date where DHM entered the hospital storage. When all bottles of a lot were completely used, technicians would record this date as the ‘finish-date.’ All information was stored in a protected electronic spreadsheet. The ‘order-date’ and ‘finish-date’ were averaged to determine the ‘use-date.’ To estimate each lot’s ‘estimated expression date,’ 365 days was subtracted from the expiration date found on DHM shipment packing slips. The number of days between the ‘use-date’ and the ‘estimated expression date’ was determined to be the storage time at the hospital level. Technicians generally used all lots in order of receipt. If the ‘finish-date’ was unavailable or not recorded, the ‘order-date’ was used to determine storage time. Storage time was estimated and plotted by the DHM lot ‘order-date’, and then averaged over a month of shipments to determine DHM storage per month, which included calculating the standard deviation per month.


Equation1. [ExpirationDateofLot−365days]=EstimatedLotExpressionDate

Equation2. [Use~date−EstimatedLotExpressionDate]=LotStoragetime(LST)

Equation3. $$\left[\frac{{\text{LST}}_{\text{Lot}1}+{\text{LST}}_{\text{Lot}2}{\text{+LST}}_{\text{Lot}3}\cdots }{\text{N}\;\text{lots}\;\text{of}\;\text{D}\;\text{Min}1\text{month}}\right]=\text{Ave}.\text{STper}\;\text{Month}$$

#### Method 2: Calculating estimated lot storage times by patient (patient-level).

One hundred VLBW infants born between 2022–2024 were prospectively followed in the first 21 days of life. Estimated storage time for each patient was first determined by recording the unique ID bottle used per day, finding the lot and expiration date of each bottle, then calculating the ‘estimated expression date’ of each bottle to estimate storage time. If two or more different bottles were used in a day, then the storage time was averaged across the bottles. Every storage time per day was then averaged over the number of days receiving DHM in the first 21 days. Storage time was determined from the expiration date using the below equation.


Equation1. [ExpirationDateofBottle−365days]=EstimatedDayofBottleExpression

Equation2. [Dayoffeeding−EstimatedDayofBottleExpression]=Storagetime(ST)

Equation3. $$\left[\frac{{\text{ST}}_{\text{Day}1}{+\text{ST}}_{\text{Day}2+}{\text{ST}}_{\text{Day}3}\cdots }{\text{N}\;\text{days}\;\text{of}\;\text{DM}}\right]=\text{Ave}.\text{STper}\;\text{Patient}$$

Mother’s milk storage conditions were not included in these equations. At 21 days of life, the receipt of any mother’s own milk (MOM) was recorded and categorized by: none/very little use, 50% or less by volume, mostly MOM, or all MOM by feeding volume.

### Statistics

The number of days fed DHM and the number of unique lots used were compared with simple linear regression and ANOVA was used for lot-level and between-group comparisons of diet at 21 days. Descriptive histograms and line plots were graphed using GraphPad Prism (v. 9.1). Significance was determined as p < 0.05.

## Results

Over 24 months, 9,810 unique bottles within 167 lots were tracked. Each lot shipped to the unit contained a median 64 [27–76], five-ounce DHM bottles (Fig. 1A). The average storage time of DHM used in the hospital was an average of 8 ± 1 months (Fig. 1B) and represented hospital-level storage time, with a minimum storage time of 119 days and maximum of 317 days at the time of arrival to the nutrition management center. Only 5% of lots received were stored less than 6 months and 17% of the lots were stored more than 9 months. Each batch of a shipment was used by 15 [10–22] days (Fig. 1C). The average storage time of DHM fluctuated per month, with mean and standard deviations presented by month (Fig. 1D). Missing data existed for 18 lots (11%).

DHM was recorded by lot and bottle number for the first 21 days of 100 DHM-eligible VLBW infants and represented patient-level measurements. The median number of lots received per infant was 3 [2–5 IQR] lots per patient (Fig. 2A). Sixty-one percent of infants received feeds of at least 50% mother’s milk feeds by 21 days. The number of unique lot exposures was proportional to the number of days receiving DHM (R^2^ = 0.726, p < 0.001) (Fig. 2B). The number of lots administered to an infant was not related to the availability of mother’s own milk intake by the infant at 21 days (Fig. 2C). The average storage duration of DHM at the time of infant feeding was 249 [238–256 IQR] days or 8.3 ± 0.6 months across all preterm infants in the study.

## Discussion

This study is the first to report the average storage time of banked DHM used in a U.S. academic children’s hospital. The average storage time of milk received in the hospital nutrition management center was 8 ± 1 months, which was consistent with the measurements of milk received by VLBW infants in the NICU. These two measurements were slightly different but correlated well, due to the rapid use of each lot, with a turn-over of 15 days. The storage time of DHM ranged from 119 to 317 days. No milk was observed to be fed beyond 12 months in this study. These results comply with current storage recommendations for frozen human milk set by both the U.S. government and HMBANA. The CDC (1) (2022) and the Academy of Breastfeeding Medicine (7) state that human milk can be safely stored up to 1 year in the deep freezer (−20°C) before feeding, but that milk stored less than 6 months is best. In our single center, DHM was stored for more than 6 months throughout the 2-year study period, and only 5% of the milk received during the study period was stored less than 6 months. Additionally, more than 17% of the lots received were stored greater than 9 months.

There are no specific recommendations for the storage of DHM designed for fragile preterm infants. While 6 months is recommended for optimal storage for home use in full-term infants, milk that was received by the hospital for the VLBW population was rarely within this timeframe. We also found that DHM storage fluctuates per month and within a single month. In this study, total storage duration of human milk fluctuated and continued to drift in one direction beyond the period of a year. Variances may be attributable to seasonal fluctuations in donation activity, processing-associated rate limiting steps, or ordering needs by the hospital.

Preterm infants received at least 3 unique lots of donor human milk, with some infants receiving up to 10 different lots. The number of different lots received was significantly associated with the number of days receiving DHM. There was no association between the number of unique lots received and the amount of mother’s milk received at 21 days of life. This finding supports the idea that receiving DHM does not subsequently impact mother’s milk intake by preterm infants.

Storage time variations may lead to regional differences in donor milk storage times, particularly from banks that follow 12-month HMBANA guidelines within the U.S. and Canada. A European study indicated that the total duration of DHM (at −18–30°C storage) was 10.3 ± 5 months, which is similar to our findings (8). The survey revealed that 90% (104/115) of European milk banks limited donations to a maximum of 6 months period or less of home storage. Preferred expiration dates vary internationally, with some countries requiring milk to have a maximum total storage of 6 months and others with more stringent requirements. Per Australian milk banking recommendations, raw milk is stored a maximum 3 months at − 20°C after expression and a maximum 3-month period post-pasteurization (9). Per milk banking guidelines in the United Kingdom, raw donor milk should not be stored more than 3 months before pasteurization to preserve essential enzymes, vitamin C, and prevent lipid degradation (10).

Human milk is affected by prolonged storage at −20°C, which is likely the scientific basis for several guidelines to suggest storing milk less than 6 months is best. Milk nutrients have been rarely studied in milk stored beyond 6 months (at −18°C). A study by Garcia-Lara et al observed decreases in fat, energy, and lactose beyond 3 months (5). Changes are not limited to macronutrient composition, but milk bioactivity is also affected. Lactoferrin, an important antiviral, antibiotic and anti-bacterial protein is significantly reduced by 46–65% in frozen milk following 6 months in storage (4, 11). The concentration of milk lactoferrin has been associated with lower sepsis rates, decreased rates of NEC, and supports neurodevelopment of growing preterm infants (3, 12, 13). Lysozyme has anti-bacterial properties against both gram-positive and gram-negative organisms, and significantly decreases over 6 months (2). The effect of pasteurization might compound these effects. It is unclear whether lysozyme activity and lactoferrin decrease after pasteurization, though these components are generally believed to be reduced by pasteurization in well-controlled studies (14, 15). Very few studies have reported milk composition and lipid stability beyond 6 months. Our laboratory found that free fatty acids continue to rise in raw human milk stored 6–12 months at −20° and can impact the structure of milk proteins (16, 17). HMBANA does not require macronutrient testing in banks; however, some banks are equipped with mid-infrared (mid-IR) transmission spectroscopy instruments to measure these factors.

Controlling variability in DHM storage may optimize its contents but shortening the recommended storage times may lead to unforeseen DHM shortages and reduced availability. Additional administrative support may be needed to streamline processing to meet demand if hospitals request milk stored less than 6 months. Excess milk waste may also be a result if changes in policy were broadly implemented without preparedness.

This study has a few limitations. It only reports storage practices within a single institution, and acquiring network level data would be more indicative of nationwide practices. However, most current hospital-based datasets do not contain the above variables in traditional electronic medical systems. A hospital-based dataset most accurately describes storage time-to-feeds, but collecting patient level data is currently time- and personnel-intensive over 2 electronic record systems. Of the 2 methods used in this study, hospital-level measures were much less burdensome than patient-level measurements. Both were in good agreement because DHM was used in chronological order of receipt.

Other limitations include that milk expiration dates are set by the earliest expressed milk in a lot, and each lot contains 3–5 other pooled donors as recommended by HMBANA to balance micronutrient composition and the diversity of human milk oligosaccharides. Traditional milk pooling has been based on the expiration date of donated milk, but more recent practices that aim to target calorie concentrations may inadvertently lead to milk stored for longer periods, due to keeping specific batches of milk for later pooling. Thus, the expiration date used in this study is only an estimate of the true storage of individual milk donations. This approach introduced systemic error by overestimating the true storage conditions of other milk samples pooled, and the robustness of the estimation could be improved if bank-level data were used.

DHM remains a vital resource to protect preterm infants and is still the best feeding choice after mother’s own milk. As the use of DHM increases, there is an opportunity to optimize the quality of human milk, specifically for the benefit of very preterm infants. Storage conditions may mostly impact high-risk patient populations. Either of the methods presented in this study are comparable, and in the context of dedicated milk research, sheds insight on the nature of DHM used in a hospital setting. Clinical studies investigating the impact of DHM on clinical outcomes should consider examining one of these novel storage variables in the future.

## Data Availability

All datasets can be made available by request to the corresponding author.

## Highlights

- The length of time that donor human milk is stored before use fluctuates over time.
- The average storage time of banked donor human milk was 8 ±1 months before use.
- Preterm infants received a median 3 unique lots of donor human milk during hospitalization.
